# Supplementary material for: MetaQTL: a package of new computational methods for the meta-analysis of QTL mapping experiments
Source: BMC Bioinformatics. 2007 Feb 8;8:49. doi: 10.1186/1471-2105-8-49 (PMC1808479; doi:10.1186/1471-2105-8-49)
Supplement: Additional File 7 — MetaQTL Package : jar file and tutorial. This Zip archive contains both the MetaQTL JAR file and the files of the tutorial. [file 1471-2105-8-49-S7.zip › org.inra.metaqtl/doc/org/metaqtl/bio/entity/CrossPopulation.html]

CrossPopulation


|  |  |  |
| --- | --- | --- |
| **Overview** \| **Package** \| **Class** \| **Use** \| **Tree** \| **Deprecated** \| **Index** \| **Help** | |  |
| **PREV CLASS**   **NEXT CLASS** | **FRAMES**    **NO FRAMES**     **All Classes** |  |
| SUMMARY: NESTED \| FIELD \| CONSTR \| METHOD | DETAIL: FIELD \| CONSTR \| METHOD |  |


---


## org.metaqtl.bio.entity Class CrossPopulation

```
java.lang.Object
  org.metaqtl.bio.entity.BioEntity
      org.metaqtl.bio.entity.BioEntityContainer
          org.metaqtl.bio.entity.IndividualContainer
              org.metaqtl.bio.entity.Population
                  org.metaqtl.bio.entity.CrossPopulation
```

**All Implemented Interfaces:**: IBioAdaptable, IBioCross, IBioEntity, IBioPopulation

---

``` public class CrossPopulation extends Population implements IBioCross ```

Class Description Here

**Author:**
:   Jean-Baptiste Veyrieras

---

**Field Summary**

**Fields inherited from class org.metaqtl.bio.entity.Population**

`generation, parents, size`

**Fields inherited from class org.metaqtl.bio.entity.BioEntityContainer**

`entities`

**Fields inherited from class org.metaqtl.bio.entity.BioEntity**

`name, parent, properties`


**Constructor Summary**

`CrossPopulation()`

`CrossPopulation(java.lang.String name,
IBioEntity parent)`

`CrossPopulation(java.lang.String name,
IBioEntity parent,
int type)`


| **Method Summary** | |
| --- | --- |
| `int` | `getCrossType()` |
| `IBioIndividual[]` | `getParents()` |
| `int` | `getType()`             There are 2 main class of entities. |
| `void` | `setCrossType(int type)` |

**Methods inherited from class org.metaqtl.bio.entity.Population**

`getBioAdapter, getGeneration, getIndividualNumber, getSize, newCrossPopulation, setGeneration, setSize`

**Methods inherited from class org.metaqtl.bio.entity.IndividualContainer**

`addIndividual, getIndividual, individuals, removeIndividual`

**Methods inherited from class org.metaqtl.bio.entity.BioEntityContainer**

`addEntity, entities, entityNumber, getEntity, removeEntity`

**Methods inherited from class org.metaqtl.bio.entity.BioEntity**

`getName, getParent, getProperties, newBioEntity, setName, setProperties`

**Methods inherited from class java.lang.Object**

`clone, equals, finalize, getClass, hashCode, notify, notifyAll, toString, wait, wait, wait`

**Methods inherited from interface org.metaqtl.bio.IBioCross**

`getGeneration, getSize`

**Methods inherited from interface org.metaqtl.bio.IBioPopulation**

`addIndividual, getIndividual, getIndividualNumber, individuals, removeIndividual`

**Methods inherited from interface org.metaqtl.bio.IBioEntity**

`getName, getParent, getProperties, setName, setProperties`

**Methods inherited from interface org.metaqtl.bio.IBioAdaptable**

`getBioAdapter`

**Constructor Detail**

### CrossPopulation

```
public CrossPopulation()
```

---


### CrossPopulation

```
public CrossPopulation(java.lang.String name,
                       IBioEntity parent)
```

**Parameters:**: `name` -: `parent` -

---


### CrossPopulation

```
public CrossPopulation(java.lang.String name,
                       IBioEntity parent,
                       int type)
```


**Method Detail**

### getCrossType

```
public int getCrossType()
```

:   **Specified by:**: `getCrossType` in interface `IBioCross`

---


### getType

```
public int getType()
```

:   **Description copied from interface: `IBioEntity`**
:   There are 2 main class of entities. The first one deals with population
    biological entity, i.e population itself and individuals. The second class
    is a representation of microscopic biological entity from genome container
    to alleles.

    :   **Specified by:**: `getType` in interface `IBioEntity` **Overrides:**: `getType` in class `Population`

---


### getParents

```
public IBioIndividual[] getParents()
```

:   **Specified by:**: `getParents` in interface `IBioCross` **Overrides:**: `getParents` in class `Population`

---


### setCrossType

```
public void setCrossType(int type)
```

:   **Parameters:**: `type` - The type to set.


---


|  |  |  |
| --- | --- | --- |
| **Overview** \| **Package** \| **Class** \| **Use** \| **Tree** \| **Deprecated** \| **Index** \| **Help** | |  |
| **PREV CLASS**   **NEXT CLASS** | **FRAMES**    **NO FRAMES**     **All Classes** |  |
| SUMMARY: NESTED \| FIELD \| CONSTR \| METHOD | DETAIL: FIELD \| CONSTR \| METHOD |  |


---
